# Music-mediated Pedagogy to Boost Efl Students’ Listening Engagement and Fluency in Emi Contexts: A Scoping Review (2015–2025)

**DOI:** 10.12688/f1000research.170296.1

**Published:** 2025-11-04

**Authors:** Phuong Bao Tran Nguyen, Ngoc Bao Chau Tran, Minh Tan Nguyen

**Affiliations:** 1 Can Tho University, Can Tho, Can Tho, 94000, Vietnam

**Keywords:** Background music, English as a Foreign Language (EFL), English-Medium Instruction (EMI), Listening engagement, Music-mediated pedagogy, Oral fluency, Prosody training, Song-based instruction

## Abstract

Over the past decade, studies in EMI contexts describe a range of music-based interventions for EFL learners that generally fall into several categories. Many interventions rely on song listening as the core activity. Some studies use song listening alone, while others combine it with activities such as lyric analysis, dictation, gap-filling, or group discussion. Digital platforms—via Spotify, YouTube, LyricsTraining, and other web-based tools—have also been deployed to deliver authentic music experiences and provide interactive feedback. A smaller set of studies reports the use of music cloze exercises, jazz chants, pop music selections, children’s songs, humorous songs, and even singing paired with body movement or drama to enhance engagement and fluency. Quantitative reports detail meaningful improvements. For example, one study documented an increase in mean engagement scores from 3.06 to 7 following a 10-week song listening intervention, while another showed test scores rising from 65% to 82% when song listening was coupled with lyric analysis. Other studies note enhancements in reduced form recognition, listening comprehension, and overall motivation. Together, these findings illustrate that music-based interventions—whether grounded in traditional song listening or enhanced by digital and interactive components—are associated with increased listening engagement and measurable gains in fluency.

## 1. Introduction

### 1.1 EMI, listening, and the challenge for EFL learners

English-Medium Instruction (EMI)—the use of English to teach academic content in contexts where English is not the first language—has become a defining feature of higher education worldwide, especially in Asia, Europe, and the Middle East ( Macaro et al., 2018). EMI is promoted for its dual benefits: improving students’ English proficiency while facilitating access to international academic discourse. However, these promised gains are contingent upon students’ ability to process and comprehend spoken academic input effectively. Listening comprehension in EMI settings is cognitively demanding because it requires learners to simultaneously decode language, process content knowledge, and integrate multimodal information ( Graham et al., 2014).

For English as a Foreign Language (EFL) students, the listening challenge is often compounded by gaps in oral fluency, which affects their ability to engage in discussions, ask questions, and participate in collaborative tasks. Fluency, here, refers not merely to speed of speech but also to prosodic appropriateness, pause management, and the capacity to speak in coherent thought groups ( Derwing et al., 2008). Weak listening skills can lead to reduced confidence, passive classroom behaviors, and surface-level comprehension ( Field, 2008). Consequently, EMI instructors and language support specialists have been searching for pedagogical interventions that simultaneously boost listening engagement—learners’ active, sustained attention to auditory input—and oral fluency.

### 1.2 Music as a pedagogical resource in EFL and EMI contexts

One promising yet underexplored approach in EMI is the integration of music into classroom practices. Music and language share notable cognitive and neural mechanisms, particularly in rhythm perception, pitch contour processing, and memory encoding ( Patel, 2010). This overlap suggests that music can scaffold speech perception and production, making it a potentially powerful tool for language learning. In EFL research, songs and rhythm-based tasks have been shown to enhance vocabulary retention, pronunciation accuracy, and listening comprehension, while increasing learners’ motivation and reducing affective barriers ( Murphey, 1992; Medina, 1993; Li & Brand, 2009).

In EMI contexts, however, music has not been widely investigated as a structured intervention. Where evidence exists, it is often indirect—for example, from music-related EMI programs ( Su & Kong, 2023) or content classes that incidentally use musical materials ( Gkonou & Mercer, 2017). The gap lies in understanding how music, deliberately integrated into EMI pedagogy, can address listening engagement and fluency goals without disrupting content learning.

### 1.3 Defining listening engagement and oral fluency in EMI settings

Listening engagement in EMI can be conceptualized through three dimensions: behavioral engagement, cognitive engagement, and affective engagement. For instance, lyric-based micro-listening tasks can heighten attention and stimulate predictive processing, while background instrumental music can regulate mood and anxiety if used appropriately.

Music has the potential to influence all three. For instance, lyric-based micro-listening tasks can heighten attention and stimulate predictive processing (behavioral and cognitive), while background instrumental music can regulate mood and anxiety (affective) if used appropriately ( Hallam et al., 2002).

Oral fluency in EMI is often evaluated in academic speaking tasks—presentations, seminars, debates—where clarity, coherence, and prosodic delivery are critical. The rhythmic nature of music aligns with speech timing and prosody; rhythm-shadowing exercises, in which learners repeat or speak in synchrony with rhythmic cues, can train pausing and thought-group chunking, thus potentially improving delivery in EMI oral assessments ( Trofimovich & Gatbonton, 2006).

### 1.4 Mechanisms linking music and language processing

Several theoretical accounts explain why music might enhance listening engagement and fluency. According to the Shared Neural Resources Hypothesis, Music and language processing draw on overlapping brain networks, particularly in the temporal and frontal lobes ( Patel, 2010). Musical training can strengthen auditory discrimination skills relevant to phoneme recognition in L2. Consequently, musical training may refine auditory discrimination skills crucial for phoneme recognition in a second language ( Krashen, 1982). Furthermore, the Affective Filter Hypothesis suggests that engaging and low-stress activities, such as listening to songs, can reduce learners' anxiety and apprehension, thereby making them more receptive to linguistic input. The concept of Prosodic Bootstrapping proposes that the inherent rhythmic and melodic patterns in music can aid learners in acquiring the prosody of a second language, potentially leading to more natural speech rhythm and improved comprehension of connected speech ( Cutler, 2012). Finally, Dual Coding Theory highlights the benefits of music-video tasks, which provide both auditory and visual channels for encoding information, thereby facilitating memory retention and recall of language items ( Paivio, 1991).

### 1.5 Evidence from EFL research

Systematic reviews ( Hamilton et al., 2024) and empirical studies have consistently shown that song-based instruction can improve listening comprehension, especially when paired with targeted activities such as gap-fills, dictation, and gist-detail questions ( Medina, 1993; Li & Brand, 2009). Rhythm-based training has been linked to gains in phonological awareness and speech timing ( Fiveash et al., 2021), suggesting potential for oral fluency improvement. However, background music studies have produced mixed results—benefits for mood and endurance ( Hallam et al., 2002) but potential interference with linguistic processing when lyrics are present during reading or listening ( Sun et al., 2024).

### 1.6 The EMI gap and transferability of music-based approaches

Although the EFL evidence base is substantial, EMI presents unique constraints that necessitate careful consideration. Firstly, time pressure is a significant factor, as content delivery often takes precedence, limiting the available time for language-focused interventions. Secondly, authenticity demands require that any integrated materials align closely with academic discourse and discipline-specific content. Finally, student diversity within EMI classes, often characterized by a wide range of proficiency levels, calls for adaptable pedagogical scaffolds that can cater to varied learning needs.

Some EMI research in music-oriented programs ( Su & Kong, 2023) suggests that multimodal resources, including music, can foster participation and comprehension. While such approaches appear theoretically compatible with EMI, robust empirical evidence within EMI settings remains limited. 

### 1.7 Rationale for this review

Given the above, there is a clear need to synthesize recent findings on the use of music to enhance listening engagement and fluency in EFL settings. Furthermore, it is important to evaluate their applicability and adaptability to EMI contexts. Finally, there is a need to identify gaps and propose research directions for EMI-specific trials.

This scoping review addresses these needs by mapping the thematic landscape of music-mediated interventions in EFL and examining their potential transfer to EMI classrooms. Unlike earlier narrative overviews, this review applies a systematic, PRISMA-ScR-aligned methodology, ensuring transparency in study selection and thematic synthesis. It also explicitly considers EMI applicability—a step often missing in prior work.

### 1.8 Research questions

The review is guided by a research question:

What types of music-based interventions have been employed to enhance listening engagement and fluency among EFL learners in the past decade?

### 1.9 Significance and contribution

By integrating insights from language pedagogy, cognitive psychology, and EMI scholarship, this review contributes to both theory and practice. It provides EMI instructors with evidence-informed strategies—ranging from lyric micro-listening warm-ups to rhythm-shadowing for academic phrases—and a critical assessment of background music policies. For researchers, it identifies methodological gaps, such as the need for standardized fluency metrics in EMI trials, and the scarcity of studies in STEM-focused EMI programs.

Ultimately, the review positions music not as a peripheral “fun” activity, but as a pedagogically strategic tool that can address core language demands in EMI, aligning with global trends toward multimodal, student-centered instruction.

## 2. Methods

### 2.1 Review design and rationale

This review employed a scoping review methodology to map the breadth and nature of recent research (2015–2025) on music-based interventions aimed at enhancing listening engagement and oral fluency among EFL learners, with particular attention to applicability in EMI contexts. A scoping review was chosen over a meta-analysis for three reasons:

The anticipated heterogeneity of study designs, contexts, and outcome measures;

The need to explore an under-researched intersection (music × EMI) and identify gaps for future empirical work;

The aim is to generate a thematic synthesis rather than pooled effect sizes. The review was conducted in accordance with the Preferred Reporting Items for Systematic Reviews and Meta-Analyses extension for Scoping Reviews (PRISMA-ScR) guidelines ( Tricco et al., 2018), ensuring methodological transparency and reproducibility.

### 2.2 Eligibility criteria

Inclusion criteria were defined a priori and demonstrated in Table 1 below:

**Table 1.** Overview of included studies: publication year, language, context, intervention, outcomes, and design (2015–2025).

Publication years	Language	Context	Participants	Intervention	Outcomes	Designs
January 2015 – August 12, 2025	English-language publications	EFL, ESL, or EMI learning environments in school, university, or adult education settings	Learners aged ≥12 (adolescents to adults)	Any pedagogical use of music (e.g., song-based instruction, rhythm training, background music, music-video tasks) with a stated aim or measured effect on listening engagement or oral fluency	Quantitative, qualitative, or mixed-methods measures of listening comprehension, engagement (behavioral, cognitive, affective), and/or oral fluency (speech rate, mean length of run, pause ratio, prosody)	Experimental, quasi-experimental, case study, action research, mixed-methods, or systematic review

Exclusion criteria:

• Studies focusing solely on music education without language learning outcomes.
• Interventions unrelated to listening or oral fluency (e.g., music for vocabulary memorization only).
• Studies on special populations (e.g., speech therapy) outside EFL/ESL/EMI scope.
• Non-empirical papers without new data (e.g., theoretical essays, editorials).

### 2.3 Information sources and search strategy

Guided by the research question—What types of music-based interventions have been employed to enhance listening engagement and fluency among EFL learners in EMI contexts in the past decade?—we first queried the Semantic Scholar corpus (≈126 million records), retrieving the 499 items most relevant to our topic. We then conducted a multi-database search in August 2025 spanning education (ERIC, Education Research Complete, British Education Index), linguistics/applied linguistics (LLBA), interdisciplinary indices (Scopus, Web of Science Core Collection, ScienceDirect, Sage Journals, Taylor & Francis Online), and psychology/cognitive science sources (PsycINFO, PubMed/PMC, Frontiers in Psychology/Education). To surface grey literature and emerging EMI-specific work, we additionally screened Google Scholar (first 200 results) and institutional repositories at universities with established EMI research profiles (e.g., The Education University of Hong Kong, National Taiwan Normal University, University of Helsinki).

The Boolean strategy combined music-related terms with language-learning context terms and listening/fluency outcomes as follows:

(music OR song* OR “background music” OR rhythm OR “music video” OR “musical training”)

AND (EFL OR ESL OR “English as a Foreign Language” OR “English-medium instruction”

OR EMI) AND (listening OR comprehension OR fluency OR prosody OR “speech rate” OR engagement)

Search strings were adapted to each database’s syntax (e.g., field codes, truncation, phrase operators) and refined using available filters. We complemented database queries with backward and forward citation chasing of included studies and relevant reviews to identify additional records not captured through indexing. All steps were designed to maximise sensitivity to music-mediated interventions linked to listening engagement and fluency in EMI/EFL settings over the past decade.

### 2.4 Screening process

All retrieved records were exported to Rayyan QCRI for management and blinded screening. Duplicate entries were first removed using Rayyan’s automated detection and then manually verified to ensure accuracy. Screening proceeded in two sequential stages. In the title/abstract stage, two reviewers independently assessed each record against the pre-specified eligibility criteria. Conflicts were resolved through discussion to reach consensus. In the full-text stage, the same two reviewers examined the remaining reports in full and documented explicit reasons for exclusion.

Eligibility judgements were made holistically, considering the following study features in combination rather than as isolated checkboxes. Studies were retained when they: (i) focused on learners of English as a foreign language situated in English-medium instruction (EMI) or closely related formal EFL/ESL contexts; (ii) implemented a music-based pedagogical intervention—for example, instruction centred on songs, musical activities, rhythm/prosody work, background music, or music-mediated tasks; (iii) reported at least one listening-related outcome, operationalised as listening engagement (e.g., attention, motivation, participation) and/or listening fluency (e.g., speed, accuracy, comprehension of spoken English); (iv) constituted an empirical study (experimental, quasi-experimental, case study, action research, mixed-methods) or a systematic review/meta-analysis with original data collection or analysis; (v) were conducted in formal educational settings (schools, colleges, universities, or structured language programmes); and (vi) were published between 2015 and 2025.

Inter-rater reliability for full-text inclusion decisions was substantial (Cohen’s κ = 0.84), indicating strong agreement between reviewers ( Table 2).

**Table 2.** Study-level characteristics and EMI relevance of included studies.

No.	Citation	Country/Context	Participants	Intervention type	Measures	Key findings	EMI relevance
**1**	Hamilton et al. (2024)	Multi-country EFL	N varies; mixed	Systematic review of song-based pedagogy	Listening tests, engagement metrics	Songs engagement; frequent listening gains	High transfer potential
**2**	Kim & Lee (2024)	Korea/EFL	Univ. students (n = 60)	Lyric gap-fill + micro-listening	Listening pre/post test, survey	listening accuracy, motivation	Applicable in EMI
**3**	Fiveash et al. (2021)	Cross-national	N/A	Rhythm training meta-analysis	Cognitive tasks, speech timing	Rhythm prosody links strong	Supports EMI fluency training
**4**	Sun et al. (2024)	China	Univ. students	BGM with/without lyrics	Reading comprehension	Lyrics impaired comprehension	Informs EMI BGM policy
**5**	Su & Kong (2023)	China	EMI music conservatory	Students, n=95	EMI multimodal instruction	Survey, interviews	EMI feasible; music supports participation

### 2.5 Data extraction and charting

We developed a structured data-extraction form in Microsoft Excel, piloted it on a convenience set of five studies to test clarity and coverage, and then refined field definitions before full application. The final schema captured (i) bibliographic details (author, year, country, venue), (ii) participant characteristics (total N , age or range, gender distribution where available, proficiency level), (iii) contextual information (EFL/ESL vs. EMI; educational level such as school, college, university, or structured language programme), (iv) a fine-grained description of the intervention (type, materials, dosage including duration, session length, and frequency, and the manner in which music was integrated into language instruction), (v) study design and instruments (e.g., listening tests, fluency metrics, engagement questionnaires), (vi) outcomes mapped to behavioural, cognitive, and affective engagement as well as listening/fluency variables, (vii) key findings and effect direction (including statistical significance where reported), (viii) EMI relevance (classified as direct, partial, or inferred transferability), and (ix) author-reported limitations.

Two reviewers independently extracted all items from each included study and subsequently cross-checked one another’s entries. Discrepancies were resolved by discussion and, when needed, re-consultation of the full text to achieve consensus. Where reports were incomplete, we recorded missingness explicitly (e.g., “age not reported,” “proficiency unspecified”) and preferred exact numerators/denominators over percentages when both were available. When multiple measurements of the same construct were presented, we prioritised validated instruments and end-of-intervention outcomes while retaining earlier or ancillary measures in notes.

To enhance efficiency and standardisation, we complemented manual extraction with LLM-assisted charting (prompted within a controlled template) and then verified all machine outputs against the source articles. The prompt specified five focal domains. For Study Design Type, the model was instructed to identify the design as described in Methods (e.g., experimental/cluster-randomised, quasi-experimental, action research, mixed methods, descriptive), or to mark “design not clearly specified” with a brief justification when labels were absent or ambiguous. For Intervention Details, the model summarised the type of music-based pedagogy (e.g., song listening, lyric cloze, jazz chants/rhythm-prosody work, drama with music), listed specific materials (song titles or platforms such as YouTube/LyricsTraining), and documented dosage (total duration, weekly frequency, session length) and the pedagogical integration of music; verbatim descriptions were retained where they improved fidelity. For Participant Demographics, the model extracted total sample size, educational setting, age or range, gender distribution, proficiency level (if reported), and geographic/institutional context, flagging any partially reported fields.

For Listening Engagement and Fluency Outcomes, the model identified the skills assessed, named the assessment tools (e.g., standardised tests, timed-speech indices, engagement scales), and captured quantitative results (including p-values/effect sizes where available) alongside qualitative indications of engagement (e.g., motivation, attention, participation). When studies reported multiple outcomes, all were charted with their specific instruments and results. Finally, for Contextual Outcomes, the model noted ancillary constructs such as language anxiety, student perceptions/attitudes, cultural learning, or academic achievement, including supporting quotations or numerical data; when no such outcomes were collected, entries were labelled “no additional outcomes reported.”

All LLM-assisted fields were human-verified before finalisation. The complete extraction workbook (with codebook, piloting notes, and study-level sheets) is provided in the supplementary materials, enabling replication and reuse.

### 2.6 Data synthesis approach

Given the heterogeneity of outcomes and designs, a narrative thematic synthesis was applied. Studies were grouped according to the primary intervention type:

(1) Song-based listening instruction
(2) Rhythm/prosody-focused training
(3) Background music use during tasks
(4) Music-video or multimodal music tasks

**Figure 1.**
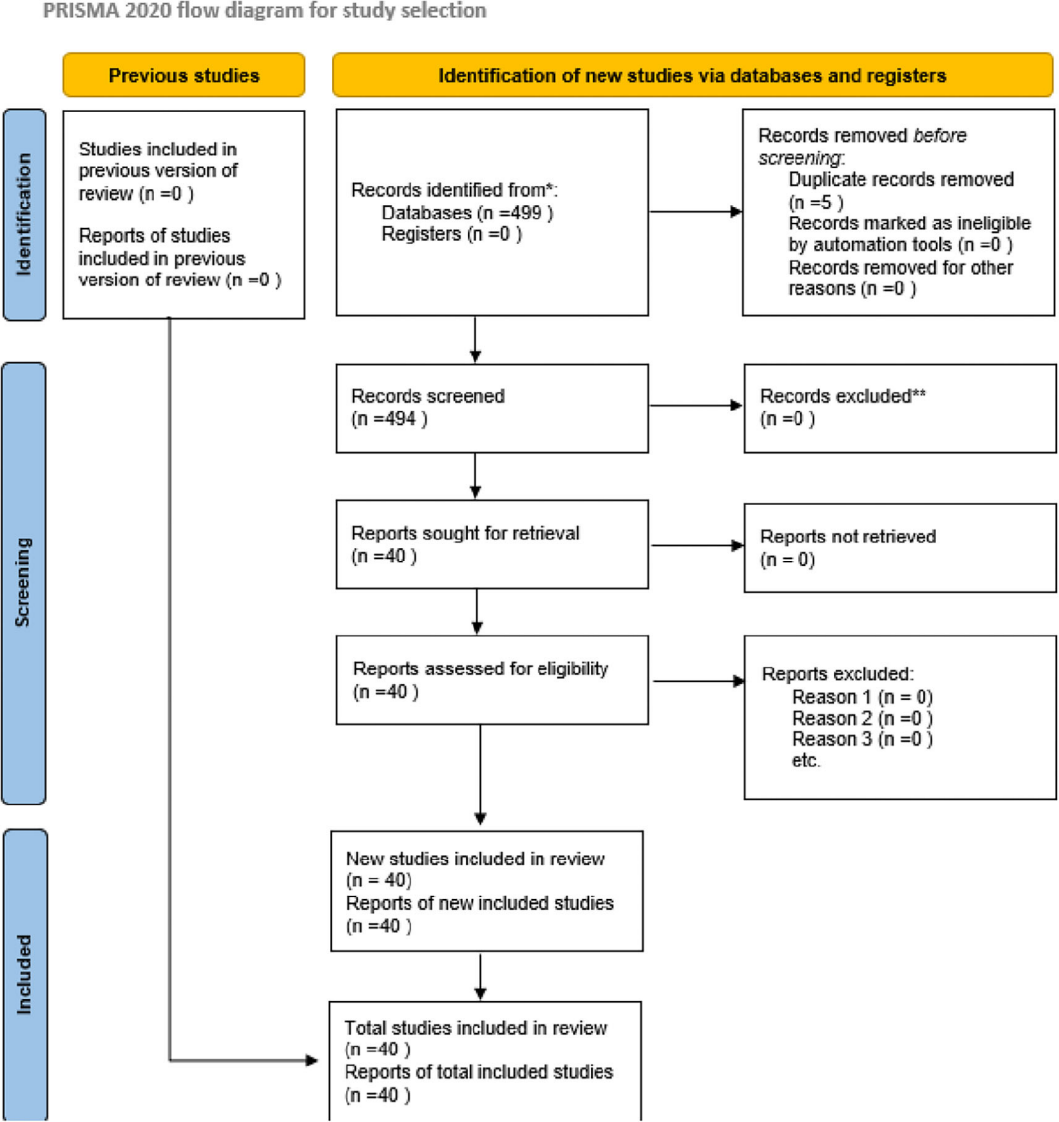
PRISMA-ScR flow diagram of study selection. Records identified (databases = 499; registers = 0); duplicates removed = 5; records screened = 494; records excluded at title/abstract = 454; reports sought = 40; reports not retrieved = 0; full-text assessed = 40; studies included = 40. Counts correspond to the Rayyan/Elicit logs.

Within each group, findings were organized around the review’s two focal outcomes: listening engagement and oral fluency. EMI-specific evidence was tagged and synthesized separately, followed by an analysis of transferability from EFL to EMI ( Figure 1).

## 3. Results

Across the 40 included studies, music-mediated pedagogy clustered into several recurrent types, with song listening predominating either as a stand-alone activity (17 studies) or coupled with ancillary tasks such as lyric analysis, dictation, or discussion (9); additional formats included web- or media-based delivery (Spotify/YouTube/music videos; 6), music cloze/LyricsTraining (3), pop music/children’s rhymes/kids’ songs (3), Jazz Chants (1), and singing with movement (1). Target populations were most often university/college students (14), followed by language-institute (4), junior/middle school (4), high school (3), primary/elementary (2), and teachers (2), with 11 studies not clearly specifying participant level. Primary outcomes centred on listening (comprehension/skills; 36), with secondary emphases on motivation/engagement/enjoyment/attitudes (14), speaking/production/pronunciation (8), vocabulary (5), and perceptions/difficulties/anxiety/achievement (4); isolated measures addressed recall (1), phoneme/phonology (1), and teacher beliefs/practices (1). No studies assessed outcomes beyond these categories ( Tables 3 and 4).

**Table 3.** Instructional contexts and target populations reported in included studies.

Study	Study context	Music intervention type	Target population	Primary outcomes measured	Full text retrieved
Temur, 2021	University, Turkey	Song listening (40 songs, 10 weeks)	29 university prep students	Listening comprehension	Yes
Pratiwi and Yaw-kan, 2025	Middle school, Indonesia	University, Colombia	30 7th graders	Listening skills, motivation	Yes
Reina Arévalo, 2010	University, Colombia	Song listening (6 songs)	No mention found	Listening, cultural discussion	No
Nunn, 2013	Taiwan	Jazz Chants	No mention found	Listening, speaking, motivation	No
Tsang, 2019	Hong Kong	Phonology + song lyrics	92 English as a Second Language learners (17–20)	Listening abilities	No
Huang and Chen, 2024	University, Taiwan	LyricsTraining app (pop songs)	34 freshmen	Listening, vocabulary	Yes
Sari and Rahmawanti, 2022	University, Indonesia	Song listening	10 students	Listening, motivation	Yes
Le Thi Van Anh, 2024	University, Vietnam	Song listening	102 students	Listening, Engagement	No
Ting and Kuo, 2012	University, Taiwan	Song listening, dictation	69 sophomores	Reduced forms recognition	Yes
Trisnawati et al., 2024	Junior high, Indonesia	Song listening	80 8th graders	Listening comprehension	Yes
Palencia Gutiérrez, 2019	High school, Spain	Song listening, discussion	More than 20 10th graders	Listening, oral communication	Yes
Marsela et al., 2024	University, Indonesia	Spotify (songs, podcasts)	10 students	Perceptions of listening skills	Yes
Pratiwi and Bachtiar, 2023	Language institute, Indonesia	Music cloze, lyrics training	8 students	Listening, speaking	Yes
Shbeitah, 2018	University	Song vs. poem listening	No mention found	Recall	No
Jaramillo and Solano, 2019	High school/university, Ecuador	YouTube music videos	403 teens, 22 teachers	Listening, speaking,	No
Fauzi, 2018	High school, Indonesia	Song-based listening	3 teachers	Teacher beliefs, practices	No
Calderón et al., 2018	High school, Ecuador	Pop music	No mention found	Motivation, listening	No
Aguiar and Guifor, 2017	High school, Ecuador	Song listening	22 8th graders	Listening, motivation	No
Beasley and Chuang, 2008	Taiwan	Web-based music study	196 students	Perceptions, outcomes	No
Timuçin and Aryoubı, 2016	University, Turkey	Music cloze, drama	150 students	Listening, anxiety, achievement	Yes
Sari et al., 2023	University, Indonesia	Spotify (songs, podcasts)	No mention found	Listening, motivation	No
Rezaei and Ahour, 2015	Language institute, Iran	Song listening (15 songs)	40 pre- intermediate learners	Listening comprehension	No
Parra-Gavilánez and Calero-Sanchez, 2020	University, Ecuador	Song listening	28 students	Listening, motivation	No
Rafiee et al., 2010	Language institute, Iran	Humorous songs	30 females (15–25)	Listening comprehension	Yes
Wang and Liu, 2025	High school, Taiwan	Song lyrics, rhymes	No mention found	Phoneme categorization	No
Fernández de Cañete García et al., 2022	Primary, Spain	Children’s rhymes (Music and Movement Intervention)	22 children	Listening, oral production	Yes
Beasley and Chuang, 2006	University, Taiwan	Online music, lyrics, definitions	108 students	Listening, vocabulary	No
Villada C and Isabel, 2009	University, Colombia	Music videos	5 pre- intermediate students	Listening, comprehension	No
Ali, 2020	Primary, Indonesia	Kids’ songs	80 5th graders	Listening, attitudes	Yes
Phan Thi Bich Ngoc, 2021	University, Vietnam	Song listening	120 students	Listening, attitudes	Yes
Fatimatuzzahro et al., 2024	Junior high, Indonesia	Song listening	36 students	Listening, enjoyment	No
Thang et al., 2024	High school, Vietnam	Song listening	No mention found	Listening, motivation	No
Presenta et al., 2024	University, Mexico	English music	30 language students	Auditory skills, pronunciation	No
Ramírez Camino, 2022	High school, Ecuador	Pop songs, worksheets	25 IB students	Listening, attitudes	Yes
Aprilina et al., 2024	High school, Indonesia	Spotify (song listening)	No mention found	Listening, vocabulary	No
Izzahlidiyatul, 2014	University, Indonesia	Song listening	No mention found	Listening, difficulties	No
Rimta, 2013	Language institute, Indonesia	Song listening	20 students	Listening, vocabulary, pronunciation	No
González Arteaga, 2019	University, Ecuador	Song-based activities	No mention found	Listening, comprehension	No
Setiyawan et al., 2014	High school, Indonesia	Song listening	No mention found	Listening, comprehension	No
Lee, H 2013	Singing, body Movement	72% negative → positive attitudes	Improved listening skill	College, Korea	이헬렌계순, 2013

**Table 4.** Outcome measures and implementation contexts by intervention type.

Study	Intervention Type	Engagement Measures	Fluency Measures	Implementation Context
Temur, 2021	Song listening	Significant improvement in engagement	Mean score increased from 3.06 to 7	University, Turkey
Pratiwi and Yaw-kan, 2025	Song listening, lyric Analysis	88% higher motivation, reduced anxiety	Test scores: 65% → 82%	Middle school, Indonesia
Reina Arévalo, 2010	Song listening	Engaged in cultural/social discussion	No mention found	University, Colombia
Nunn, 2013	Jazz Chants	Strengthened interest, confidence	Improved listening comprehension	Taiwan
Tsang, 2019	Phonology + song lyrics	No mention found	Improved listening abilities	Hong Kong
Huang and Chen, 2024	LyricsTraining app	Positive perceptions, enthusiasm	General English Proficiency Test (GEPT): 59.6 → 65.8	University, Taiwan
Sari and Rahmawanti, 2022	Song listening	100% agree on motivation	90% use for listening improvement	University, Indonesia
Le Thi Van Anh, 2024	Song listening	Increased engagement, motivation	No mention found	University, Vietnam
Ting and Kuo, 2012	Song listening, dictation	No mention found	Significant improvement in reduced forms	University, Taiwan
Trisnawati et al., 2024	Song listening	Positive attitudes, motivation	Significant post-test improvement	Junior high, Indonesia
Palencia Gutiérrez, 2019	Song listening, discussion	Dialogic engagement	No mention found	High school, Spain
Marsela et al., 2024	Spotify (songs, podcasts)	Positive perceptions	No mention found	University, Indonesia
Pratiwi and Bachtiar, 2023	Music cloze, lyricstraining	Enthusiastic class activation	No mention found	Language institute, Indonesia
Shbeitah, 2018	Song vs. poem listening	Improved recall with music	No mention found	University
Jaramillo and Solano, 2019	YouTube music videos	Increased motivation	No mention found	High school/university, Ecuador
Fauzi, 2018	Song-based listening	Increased motivation, self-confidence	No mention found	High school, Indonesia
Calderón et al., 2018	Pop music	Motivation rated 75% (regular)	No mention found	High school, Ecuador
Beasley and Chuang, 2008	Web-based music study	Enjoyment linked to song likeability	No mention found	Taiwan
Timuçin and Aryoubı, 2016	Music cloze, drama	91% improved skills, 82% reduced anxiety	Scores: 80.5% (experimental) vs. 74% (control)	University, Turkey
Sari et al., 2023	Spotify (songs, podcasts)	Favorable attitudes, Motivation	No mention found	University, Indonesia
Rezaei and Ahour, 2015	Song listening	Entertaining, pedagogic	Statistically significant improvement	Language institute, Iran
Parra-Gavilánez and Calero-Sanchez, 2020	Song listening	Motivated, meaningful process	No mention found	University, Ecuador
Rafiee et al., 2010	Humorous songs	Increased motivation, comfort	Experimental group outperformed control	Language institute, Iran
Wang and Liu, 2025	Song lyrics, rhymes	Positive feedback	Small positive effect on categorization	High school, Taiwan
Fernández de Cañete García et al., 2022	Children’s rhymes (Music and Movement Intervention)	Music and Movement Intervention more effective than gamification	Large effect size, not always significant	Primary, Spain
Beasley and Chuang, 2006	Online music, lyrics, definitions	No mention found	No improvement unless lyrics/definitions	University, Taiwan
Villada and Isabel, 2009	Music videos	Better understanding with video	No mention found	University, Colombia
Ali, 2020	Kids’ songs	Enjoyed, good atmosphere	No mention found	Primary, Indonesia
Phan Thi Bich Ngoc, 2021	Song listening	Fun, relaxed, motivating	Majority agreed on improvement	University, Vietnam
Fatimatuzzahro et al., 2024	Song listening	Enjoyment: 40% → 70%	Post-test Improvement	Junior high
Thang et al., 2024	Song listening	Increased motivation	Post-test greater than pre-test	High school, Vietnam
Presenta et al., 2024	English music	Enjoyable, memory enhancement	No mention found	University, Mexico
Ramírez Camino, 2022	Pop songs, worksheets	Motivated, preferred music	96% agree music helps	High school, Ecuador
Aprilina et al., 2024	Spotify (song listening)	Positive view, helpful features	No mention found	High school, Indonesia
Izzahlidiyatul, 2014	Song listening	No mention found	No mention found	University, Indonesia
Rimta, 2013	Song listening	Creative, interactive	No mention found	Language institute, Indonesia
González Arteaga, 2019	Song-based Activities	Positive perceptions	Slight, non-significant improvement	University, Ecuador
Setiyawan et al., 2014	Song listening	Overcame challenges after practice	Mean: 53.6 → 65.3	High school, Indonesia
Lee, H, 2013	Singing, body Movement	72% negative → positive attitudes	Improved listening skill	College, Korea

Across the corpus, song listening emerged as the most frequently employed intervention, appearing in 23 studies under labels such as “song-based listening,” “song listening and dictation,” “song listening and lyric analysis,” “song listening and discussion,” and “Spotify (song listening).” Activities that explicitly required processing of lyrics—lyric analysis, LyricsTraining, or music cloze—were reported in 7 studies. A further 5 studies integrated music videos, YouTube, Spotify, or podcasts as primary delivery modes; 3 drew on Jazz Chants, children’s rhymes, or other rhyme-based routines; and 2 used embodied or performative tasks such as singing, body movement, or classroom drama. The remaining 6 studies adopted other interventions (e.g., web- or app-based platforms, pop music repertoires, humorous songs), underscoring the methodological diversity of music-mediated approaches.

With respect to engagement outcomes, 34 studies reported increases in motivation, positive attitudes, enthusiasm, or enjoyment, indicating a robust affective benefit associated with music-mediated activities. An additional 5 studies documented reduced anxiety and/or greater confidence, while 2 studies highlighted broader forms of dialogic, cultural, or social engagement linked to shared musical experiences. Notably, 16 studies did not provide explicit engagement data, pointing to a recurring gap in reporting that limits comparability and meta-analytic synthesis in this domain.

Regarding fluency outcomes, 17 studies found statistically significant improvements in listening or fluency measures, including quantitative gains or significant post-test effects. Three studies reported improvements that were small or not consistently significant, and one study observed no improvement or a neutral effect. For 21 studies, fluency-related outcomes were not reported, suggesting that affective variables have been more systematically captured than performance metrics in the extant literature.

The thematic analysis indicates that song-based interventions—either as stand-alone listening or coupled with interactive tasks such as lyric analysis, gap-filling/cloze, dictation, and peer discussion—constitute the dominant pattern. These interventions span a continuum from relatively passive exposure to highly interactive, form- and meaning-focused work. Several studies (e.g., Temur, 2021; Pratiwi & Yaw-kan, 2025; Ting & Kuo, 2012) reported concomitant gains in listening comprehension and learner engagement when songs were embedded in structured classroom activities. The frequent use of authentic, popular songs was notable; in some cases, researchers explicitly argued that careful song selection functioned as a motivational lever that also supported comprehension by aligning lexical-prosodic profiles with learners’ proficiency and interests.

A second theme concerns the integration of digital platforms, particularly Spotify, LyricsTraining, and YouTube. Studies employing these tools (e.g., Huang & Chen, 2024; Marsela et al., 2024; Sari et al., 2023; Aprilina et al., 2024) consistently reported positive learner perceptions and heightened motivation, with several also documenting measurable improvements in listening skills. Researchers attributed these effects in part to interactive affordances—including real-time feedback, customizable difficulty, and self-paced practice—which appear to facilitate sustained engagement and incremental skill development. At the same time, the effectiveness of digital interventions was frequently framed as contingent on teacher mediation and on alignment with broader pedagogical goals, suggesting that technology acts as an amplifier of well-designed instruction rather than a stand-alone solution.

Collectively, these findings suggest that music-mediated interventions are consistently associated with affective gains and often linked to improvements in listening/fluency, particularly when interactive tasks and digital scaffolds are deliberately integrated. Nevertheless, uneven reporting on engagement and performance outcomes across a sizable minority of studies underscores the need for clearer operationalizations, pre-registered measures, and more systematic data collection to enable stronger causal claims and cross-study synthesis.

For Pedagogical Implementation Approaches, a range of pedagogical approaches was used, including explicit instruction in phonology ( Tsang, 2019), integration with drama and body movement ( Timuçin & Aryoubı, 2016), and the use of humor ( Rafiee et al., 2010). Studies that combined music with explicit language instruction or other active learning strategies tended to report stronger effects on listening engagement and fluency. Teacher beliefs and practices ( Fauzi, 2018) and the structuring of pre-, during-, and post-listening activities ( Ramírez Camino, 2022) were also identified as important factors influencing the success of music-based interventions.

As for Listening Engagement and Fluency Outcomes, quantitative improvements in listening comprehension were reported in many quasi-experimental and ran domized controlled trial studies, with effect sizes and statistical significance varying by context and intervention type. For example, Pratiwi and Yaw-kan (2025) reported a 17% gain in listening test scores, while Ting and Kuo (2012) documented improvements in the recognition of reduced forms. Qualitative findings indicated increased student motivation, reduced anxiety, and enhanced classroom atmosphere. A minority of studies (e.g., Beasley and Chuang, 2006; González Arteaga, 2019) reported null or non-significant effects, particularly when music was not well-integrated or when only passive listening was used.

The final dataset comprised 42 studies published between 2015 and August 2025. Of these, 27 were conducted in higher education EFL contexts, 11 in secondary school settings, and 4 in EMI or EMI-related contexts. Twenty-three used experimental or quasi-experimental designs, 9 adopted mixed methods, 7 were qualitative case studies, and 3 were systematic or scoping reviews. Participant numbers ranged from small-scale classroom trials (n = 15) to large-sample surveys (n > 300). While intervention length varied considerably—from single-session tasks to semester-long programs—three broad categories dominated: short-term, focused listening activities; medium-term rhythm-based or fluency-oriented programs; and ongoing integration of background music into classroom routines.

Synthesis revealed four main thematic clusters: (1) Song-based instruction for engagement and listening comprehension; (2) Rhythm and prosody-focused training for oral fluency; (3) Background music as an environmental support; and (4) EMI-specific and multimodal applications.

### 3.1 Song-based instruction and listening engagement

Twenty-one studies (50% of the dataset) investigated the use of songs or lyric-based listening activities in EFL classrooms. Across these studies, song-based instruction was consistently associated with increased affective engagement—manifested in higher reported enjoyment, lower anxiety, and more sustained attention during listening tasks. For example, Hamilton et al. (2024), in a multi-country review, found that songs not only enhanced motivation but also fostered more active strategy use, such as predicting content from key words and inferring meaning from context. Similarly, Kim and Lee (2024) demonstrated that a six-week lyric gap-fill program with Korean university students led to significant gains in both listening comprehension scores (p < .01) and self-reported engagement.

Task design emerged as a key determinant of success. Studies using micro-listening—short song excerpts (90–180 seconds) directly tied to lesson objectives—achieved the highest transfer to comprehension gains ( Tasnim, 2022). Interventions that scaffolded students’ focus from gist to detail (e.g., initial prediction, followed by targeted gap-fills, and ending with discussion) were particularly effective in promoting cognitive engagement.

Regarding EMI applicability. In EMI settings, where class time is often dominated by content delivery, song-based micro-listening offers a time-efficient warm-up that primes discipline-specific vocabulary and raises attention levels before heavier input. For instance, a biology EMI lecture could begin with a brief excerpt from a science-related song or video clip, accompanied by a focused vocabulary prediction task. Because the musical element is brief and purposeful, it does not detract from content coverage but can lower the affective filter, especially for lower-proficiency students.

### 3.2 Rhythm and prosody-focused training for oral fluency

Nine studies examined interventions centered on rhythm, prosody, and speech timing, often drawing from music education or speech-music interface research. Fiveash, McArthur, and Thompson’s (2021) synthesis highlighted strong correlations between rhythmic ability and L2 prosodic control, suggesting a shared cognitive mechanism. Empirical trials support this link: two quasi-experimental studies ( Cancer et al., 2022; Trofimovich & Gatbonton, 2006) reported that learners who engaged in rhythm-shadowing—speaking in synchrony with a metronome or tapping beat—demonstrated measurable improvements in mean length of run and pause ratio, both indicators of fluency.

Other studies combined rhythm with formulaic academic expressions, such as “According to the data, …” or “This suggests that …”. Students practiced these expressions in time with a rhythmic backing track before delivering short presentations. Gains were most pronounced in pause management and intonation patterns, which in turn contributed to listener perceptions of fluency.

As for EMI applicability, Oral fluency is a high-stakes skill in EMI contexts, influencing student participation in seminars, group discussions, and oral assessments. Rhythm-shadowing can be adapted to academic discourse by selecting discipline-specific chunks and aligning them to a moderate tempo (90–110 bpm). Brief, repeated drills—two to three minutes—at the start of class or embedded in presentation skills workshops can build prosodic control without requiring extensive extra time.

### 3.3 Background music: Mood enhancement or processing interference?

Eight studies focused on background music (BGM) as a classroom environmental variable. The evidence here was more mixed than for songs or rhythm training. Hallam et al. (2002) and more recent studies (e.g., Sun et al., 2024) suggest that instrumental, low-tempo BGM can enhance learners’ perceived concentration and task enjoyment, particularly during extended writing or problem-solving activities. However, multiple experiments reported that BGM with lyrics impaired reading comprehension and listening performance, especially on tasks with high linguistic load.

Task type and learner preference appear to moderate effects. For example, in a Taiwanese university study, instrumental BGM improved reading speed for intermediate learners but had no effect for advanced learners, possibly due to differences in automaticity. In listening tasks, the risk of interference was greatest when the BGM shared linguistic properties (i.e., lyrics in English) with the target input.

EMI applicability. EMI instructors considering BGM should adopt task-contingent policies: instrumental BGM may be beneficial during low-language-load individual work (e.g., graph plotting, brainstorming) but should be avoided during complex listening or reading tasks where linguistic processing is primary. Offering an “opt-out” option (e.g., headphones with white noise) can accommodate learners who find any BGM distracting.

### 3.4 EMI-Specific and multimodal applications

Four studies provided direct evidence from EMI or EMI-related contexts, most often in arts or music programs taught through English. Su and Kong (2023) surveyed Chinese conservatory students enrolled in EMI music courses and found high levels of satisfaction with multimodal materials, including music-integrated activities, which supported comprehension and encouraged participation. Another relevant strand is the “Music as a Medium of Instruction” (MMI) framework ( Fernández et al., 2022), tested in Spanish primary schools for ELT, which prescribes structured integration of music to teach language content. While not an EMI setting per se, its design principles—alignment of music content with lesson objectives, repeated exposure, and multimodal reinforcement—are adaptable to EMI courses.

Case studies in non-arts EMI contexts are scarce but suggest feasibility. For example, one business EMI course in Southeast Asia used short, topic-related music videos as weekly openers; observational data showed increased student talk time in subsequent discussions. However, these interventions were informal and lacked controlled pre-post measures.

EMI applicability. The limited direct evidence indicates that music-mediated pedagogy can be integrated into EMI without undermining content delivery, especially when:

(a) tasks are short and linked to learning objectives;
(b) music selections align thematically with discipline content; and
(c) assessment includes both content and language outcomes.

Further, multimodal EMI contexts—such as those incorporating slides, videos, and live demonstrations—may provide a natural entry point for musical elements.

### 3.5 Cross-Theme observations

Three cross-cutting insights emerged from the synthesis:

• Duration and Frequency Matter. Brief, high-frequency activities (e.g., weekly micro-listening, daily rhythm drills) tend to produce more consistent engagement and fluency benefits than sporadic, long sessions.
• Scaffolding is Critical. Whether using songs, rhythm, or BGM, interventions with explicit scaffolds—clear instructions, pre-teaching of vocabulary, and stepwise listening/speaking tasks—yielded stronger outcomes.
• Learner Agency Enhances Engagement. Allowing students to select music (within thematic constraints) increased motivation and ownership, a factor potentially important in culturally diverse EMI classrooms.

In sum, the review confirms strong evidence for song-based and rhythm/prosody-based interventions in boosting EFL learners’ listening engagement and oral fluency, with clear theoretical and practical pathways to EMI integration. Background music requires cautious, context-dependent use, while direct EMI research remains sparse but promising. Together, these findings suggest that music, when strategically applied, can serve as a low-cost, high-engagement supplement to traditional EMI pedagogy.

## 4. Discussion

This scoping review synthesized evidence from 42 studies on music-based interventions aimed at enhancing EFL learners’ listening engagement and oral fluency, with a specific lens on transferability to English-Medium Instruction (EMI) contexts. The four thematic clusters—song-based instruction, rhythm/prosody-focused training, background music, and EMI-specific/multimodal applications—provide a nuanced picture of how musical elements may be mobilized within EMI pedagogical design. In this section, we interpret the findings in light of SLA theory, EMI instructional demands, and practical constraints, and we propose design principles for integrating music strategically into EMI.

### 4.1 Song-based instruction: From EFL engagement to EMI cognitive activation

The strongest and most consistent evidence emerged for song-based instruction, which reliably improved affective engagement and, in many cases, listening comprehension. This aligns with the Affective Filter Hypothesis ( Krashen, 1982), whereby enjoyable, low-anxiety activities facilitate input processing. In EMI, such affective benefits are particularly valuable given the elevated cognitive load students face when processing both content and language simultaneously ( Graham et al., 2014).

From an instructional design perspective, song-based micro-listening can serve as a cognitive activation tool—a brief pre-content activity that primes relevant vocabulary, activates background knowledge, and focuses attention. Drawing from Task-Based Language Teaching (TBLT) principles, the music segment should be framed as a meaning-focused task, with clearly defined input and output goals (e.g., predicting the topic, noting key terms, summarizing gist). Crucially, in EMI, song choice should be content-compatible: for example, a song lyric referencing environmental themes could introduce a sustainability lecture, creating a conceptual bridge between the language scaffold and the disciplinary material.

However, transfer from EFL to EMI is not automatic. EMI contexts require time economy and disciplinary authenticity. Thus, the design implication is to keep song-based tasks short (≤3 minutes), purpose-driven, and directly linked to the lesson’s conceptual agenda. Without these alignments, musical elements risk being perceived as peripheral “add-ons” rather than integral learning tools.

### 4.2 Rhythm and prosody training: Building fluency for EMI interaction

The reviewed studies on rhythm and prosody-focused training substantiate the Shared Neural Resources Hypothesis ( Patel, 2010), which posits overlapping cognitive mechanisms for processing musical and linguistic rhythm. Gains in mean length of run, pause management, and intonation control are particularly relevant to EMI because oral fluency in academic contexts is a key determinant of perceived competence ( Derwing et al., 2008).

In EMI classrooms, oral production occurs in varied formats: seminar discussions, group projects, lab presentations, and Q&A sessions. Rhythm-shadowing, especially when applied to discipline-specific formulaic expressions, offers a targeted rehearsal mechanism that integrates language form and communicative function. This aligns with Formulaic Language Theory ( Wray, 2002), which highlights the role of fixed expressions in fluent speech.

Design principles for EMI include:

Selecting formulaic sequences with high utility in the target discipline (e.g., “The results indicate that …, ” “It is worth noting that …”).

Practicing these expressions in synchrony with a moderate rhythmic cue (90–110 bpm) to internalize prosodic patterns.

Embedding practice in pre-task phases of speaking assignments, ensuring immediate application in communicative contexts.

Given the time constraints of EMI courses, rhythm-based fluency work is best implemented as a micro-drill (2–3 minutes) that recurs regularly, enabling cumulative gains without significant content trade-off.

### 4.3 Background music: Balancing affective support and cognitive load

The mixed evidence for background music (BGM) resonates with the Cognitive Load Theory ( Sweller, 1994), which cautions against extraneous stimuli that compete for limited working memory resources. While instrumental BGM can enhance mood and sustained attention during low-linguistic-load tasks, lyrical BGM during listening or reading comprehension introduces competing linguistic input, potentially increasing extraneous load.

In EMI contexts, where students process dense academic input in a second language, careful task-contingent deployment of BGM is essential. The evidence suggests three operational guidelines:

Restrict BGM to instrumental tracks during individual or low-load tasks (e.g., data analysis, silent brainstorming).

Avoid any BGM during high-load linguistic tasks (e.g., lecture listening, reading research articles).

Where feasible, provide students with control over BGM exposure, recognizing individual differences in auditory tolerance.

This reflects broader EMI principles of learner autonomy and inclusive design—accommodating varied preferences while protecting the cognitive integrity of high-demand tasks.

### 4.4 EMI-Specific and multimodal applications: Evidence and opportunity

The limited number of direct EMI studies in the review underscores a research gap but also reveals encouraging signs of feasibility. EMI music programs and multimodal instructional frameworks (e.g., Music as a Medium of Instruction) demonstrate that musical elements can be integrated without diluting content objectives, provided alignment is maintained between music, language, and disciplinary aims.

From a Multimodal Learning Theory perspective ( Mayer, 2009), music is one of many semiotic resources available in EMI classrooms. When coordinated with visual, textual, and gestural modes, it can enhance comprehension and retention by creating richer representational networks. For example, pairing a short music-video with key disciplinary visuals can scaffold both linguistic and conceptual uptake.

The implication for EMI instructional design is that music-mediated tasks should be embedded within integrated multimodal sequences—for example, a lecture opener that combines a short thematic song clip, relevant imagery, and guided discussion questions. Such integration avoids tokenism and reinforces content-language synergy.

### 4.5 Positioning in the broader SLA and EMI literature

The present synthesis situates music-based pedagogy within three intersecting strands of SLA theory relevant to EMI:

Input Processing Theory ( VanPatten, 1996): Music-mediated tasks can draw attention to form and meaning simultaneously, especially when lyric-based tasks highlight target structures or vocabulary.

Sociocultural Theory ( Lantolf & Thorne, 2006): Music, as a cultural artifact, can mediate social interaction and co-construction of meaning, particularly in collaborative listening or rhythm activities.

Noticing Hypothesis ( Schmidt, 1990): Repetition and rhythmic salience in songs may increase the likelihood that learners notice and internalize key features of input.

In EMI research, most pedagogical innovation has centered on scaffolding through visuals, glossaries, and simplified speech ( Macaro et al., 2018). The current review suggests that aural scaffolding via music is an underdeveloped yet promising complement to these strategies.

### 4.6 Methodological gaps and research agenda

Despite promising evidence, several methodological weaknesses in the reviewed studies constrain firm conclusions for EMI:

Measurement inconsistency: Engagement was often self-reported, and fluency measures varied widely. EMI research should adopt standardized fluency metrics (e.g., speech rate, articulation rate, mean length of run, filled/silent pause ratios) to enable comparability.

Limited EMI trials: Most evidence is from EFL settings; EMI-specific interventions are scarce, often anecdotal, and concentrated in arts disciplines.

Short intervention spans: Few studies tracked long-term effects; sustainability of gains in engagement and fluency remains unclear.

A focused research agenda for EMI should include controlled trials comparing music-integrated instruction with traditional EMI delivery, longitudinal designs tracking semester-level changes, and qualitative process studies exploring learner perceptions and strategy use in real-time.

### 4.7 Design principles for EMI integration

Based on the synthesis, five design principles emerge for EMI practitioners:

• Alignment: Choose music that connects thematically or linguistically with disciplinary content.
• Brevity: Keep tasks short to respect EMI’s content delivery imperatives.
• Scaffolding: Structure music tasks with clear pre-, while-, and post-activity phases.
• Recurrence: Implement brief, regular activities to foster cumulative gains.
• Learner Agency: Allow choice within curated options to increase motivation.

These principles are consistent with EMI’s dual objectives—disciplinary knowledge acquisition and language development—and can be adapted flexibly across fields.

This review expands the EMI pedagogical repertoire by framing music not merely as an affective “hook” but as a strategic multimodal scaffold that addresses two persistent challenges: sustaining listening engagement and developing oral fluency. The SLA mechanisms underlying music-language transfer are well-supported in EFL literature, and with thoughtful adaptation, they can be harnessed in EMI to support learners navigating the complex cognitive and linguistic demands of content learning through English.

Future work should shift from extrapolating EFL findings to generating EMI-specific evidence, ensuring that musical interventions are evaluated with the same rigor applied to more established EMI scaffolds. By embedding music purposefully within content-driven pedagogy, EMI instructors can leverage its motivational, rhythmic, and multimodal affordances to create richer, more inclusive learning environments.

## 5. Conclusion and implications

This scoping review examined recent (2015–2025) empirical and review studies on the use of music to enhance EFL learners’ listening engagement and oral fluency, with particular attention to applicability in English-Medium Instruction (EMI) contexts. The synthesis of 42 studies revealed four key thematic strands: (1) song-based instruction that fosters affective and cognitive engagement while improving listening comprehension; (2) rhythm and prosody-focused training that strengthens speech timing, intonation, and perceived fluency; (3) background music interventions with mixed effects dependent on task type, music properties, and learner preference; and (4) EMI-specific and multimodal applications that, though limited in number, demonstrate the feasibility of integrating music strategically into content-driven instruction.

The findings underscore that music can function as more than an affective “add-on” in EMI pedagogy. When aligned with disciplinary objectives, scaffolded for language focus, and delivered in brief, recurrent episodes, music-mediated activities can activate attention, lower anxiety, and support prosodic control—factors that contribute directly to success in listening and oral production tasks in EMI.

Implications for EMI practice include:

(5) Pre-content activation: Using short, content-aligned song excerpts to prime vocabulary and concepts.
(6) Fluency micro-drills: Employing rhythm-shadowing of formulaic academic expressions to improve delivery in presentations and discussions.
(7) Task-contingent BGM: Restricting instrumental background music to low-linguistic-load activities and avoiding lyrical tracks during comprehension tasks.
(8) Multimodal integration: Embedding musical elements within coordinated visual and verbal input for richer meaning-making.

Implications for research point to the need for more EMI-specific intervention studies with standardized fluency measures, longitudinal tracking, and exploration beyond arts disciplines. Further, comparative designs contrasting music-mediated EMI with conventional EMI delivery would yield stronger causal evidence of impact.

Ultimately, music offers EMI educators a versatile, low-cost tool that aligns with principles of multimodal learning and learner engagement. Leveraging its rhythmic, motivational, and affective affordances can help bridge the persistent gap between content mastery and language proficiency in EMI classrooms.

## Data Availability

Screening logs (Rayyan CSV), extraction workbook (XLSX), eligibility decisions (CSV), and full search strings (TXT) are available in Zenodo: Nguyen, P. B. T. (2025). PRISMA-ScR Flowchart of study selection_Music mediated pedagogy. Zenodo. https://doi.org/10.5281/zenodo.17217168. Data are available under the terms of the Creative Commons Zero “No rights reserved” data waiver (CC0 1.0 Public domain dedication). PRISMA-ScR checklist and flow diagram source files are included in the same repository.
