# Supplementary material for: Music-mediated Pedagogy to Boost Efl Students’ Listening Engagement and Fluency in Emi Contexts: A Scoping Review (2015–2025)
Source: F1000Res. 2025 Nov 4;14:1207. [Version 1] doi: 10.12688/f1000research.170296.1 (PMC12961287; doi:10.12688/f1000research.170296.1)
Supplement: Peer review history

# Reviewer report: Reviewer response for version 1

**DOI:** 10.5256/f1000research.187738.r462513

**Published:** 2026-03-04

**Authors:** Ramanda Rizky

**Affiliations:** 1 Universitas Lancang Kuning, Pekanbaru, Riau, Indonesia

Review Report

**1. Summary of the Article**

 The article *“Music-mediated Pedagogy to Boost EFL Students’ Listening Engagement and Fluency in EMI Contexts: A Scoping Review (2015–2025)”* presents a systematic scoping review of empirical and review studies examining the use of music-based pedagogical interventions to enhance listening engagement and oral fluency among EFL learners, with a specific focus on transferability to English-Medium Instruction (EMI) contexts.

 Using a PRISMA-ScR–aligned methodology, the authors synthesize findings from 42 studies published between 2015 and 2025 across diverse educational settings. The review categorizes interventions into four main themes: song-based instruction, rhythm and prosody-focused training, background music use, and EMI-specific or multimodal applications. The article integrates insights from second language acquisition theory, cognitive psychology, and EMI research to explain underlying mechanisms and pedagogical relevance.

 Overall, the review positions music not as an ancillary motivational tool but as a theoretically grounded, multimodal scaffold capable of supporting core EMI demands, particularly sustained listening engagement and oral fluency development.

**2. Evaluation of Key Review Questions**

*a. Is the topic discussed comprehensively in the context of the current literature?*

**Answer: Yes**

 The topic is addressed comprehensively. The review draws on a broad and up-to-date body of literature, including empirical studies, systematic reviews, and foundational theoretical works. It successfully situates music-mediated pedagogy within established SLA frameworks (e.g., Affective Filter Hypothesis, Shared Neural Resources Hypothesis, Dual Coding Theory) and connects these perspectives to contemporary EMI challenges.

 The time frame (2015–2025), multi-database search strategy, and transparent inclusion criteria ensure wide coverage of relevant research. Importantly, the authors do not restrict themselves to positive findings but also incorporate studies reporting mixed or null effects, particularly regarding background music. This balanced synthesis strengthens the article’s comprehensiveness and credibility.

*b. Are all factual statements correct and adequately supported by citations?*

**Answer: Yes (with minor caveats)**

 Most factual statements are accurate and appropriately supported by citations. Quantitative claims regarding improvements in engagement, listening comprehension, or fluency are consistently attributed to specific studies. Theoretical assertions are grounded in well-established literature rather than speculative interpretations.

 Where direct EMI evidence is limited, the authors clearly signal inferential reasoning and avoid presenting extrapolated claims as definitive. This transparency is methodologically sound and appropriate for a scoping review.

 A minor limitation is that some descriptive claims (e.g., “consistently associated with increased engagement”) aggregate studies with heterogeneous measures. While this does not invalidate the claims, clearer signaling of variability in measurement quality would further strengthen evidential precision.

*c. Are the conclusions appropriate in the context of the current research literature?*

**Answer: Yes**

 The conclusions are well calibrated to the strength of the existing evidence. Rather than asserting strong causal effects, the authors emphasize patterns, tendencies, and pedagogical potential. This is consistent with both the scoping review design and the uneven empirical base in EMI-specific research.

 The conclusions appropriately highlight three key points supported by the literature:

1. music-based interventions reliably enhance affective and behavioral engagement;
2. listening and fluency gains are frequent but context- and design-dependent;
3. EMI transferability is theoretically plausible but empirically underexplored.

The recommendations for practice and future research are cautious, evidence-informed, and aligned with contemporary EMI scholarship.

**3. Points Requiring Improvement or Clarification**

 Although no major scientific flaws were identified, the following points should be addressed to further strengthen the article’s scientific rigor:

1. *Clarification of Evidence Weight*
  While the review is transparent, the conclusions occasionally treat engagement and performance outcomes with similar rhetorical emphasis. A clearer differentiation between strongly supported outcomes (engagement) and moderately supported outcomes (fluency) would enhance analytical precision.
2. *Operational Consistency*
  The review notes variability in how engagement and fluency are measured across studies. This issue is discussed but could be more explicitly reflected in the conclusions to avoid any perception of overgeneralization.
3. *EMI-Specific Evidence Delimitation*
  The article appropriately acknowledges the scarcity of EMI-specific studies; however, an explicit summary table distinguishing EFL-based evidence from EMI-based evidence in the conclusion section would further reinforce transparency and help readers interpret transferability claims.

**4. Points That Must Be Addressed to Ensure Scientific Soundness**

 The article is already scientifically sound. However, to strengthen its robustness and preempt potential reviewer concerns, the authors should:

- Explicitly restate in the conclusion that **most empirical evidence derives from EFL rather than EMI contexts**, and that EMI implications remain provisional.
- Slightly temper any generalized phrasing related to fluency gains by foregrounding the variability of outcome measures.
- Reinforce methodological reflexivity by linking identified limitations more directly to the scope of the conclusions.

These refinements would enhance clarity rather than correct substantive flaws.

**5. Overall Assessment**

 This is a **high-quality, theoretically grounded, and methodologically transparent scoping review**. It makes a meaningful contribution to EFL and EMI scholarship by synthesizing a fragmented literature and articulating a coherent pedagogical rationale for music-mediated instruction. With minor refinements focused on evidential weighting and EMI delimitation, the article is well positioned to be indexed and peer review.

Is the review written in accessible language?

Yes

Are all factual statements correct and adequately supported by citations?

Yes

Are the conclusions drawn appropriate in the context of the current research literature?

Yes

Is the topic of the review discussed comprehensively in the context of the current literature?

Yes

Reviewer Expertise:

As a researcher and academician, my expertise lies primarily in educational research and applied pedagogy, with a focus on teaching–learning processes, instructional design, and empirical inquiry in formal education contexts. This background enables me to evaluate how effectively the article frames its research problem, integrates established theories of learning and second language acquisition, and situates music-mediated pedagogy within EFL and EMI settings.I also have strong competence in educational research methodology, particularly in assessing systematic and scoping reviews. I am able to examine the appropriateness of the PRISMA-ScR approach, the clarity of inclusion criteria, the rigor of data synthesis, and the alignment between evidence and conclusions.In addition, my experience in educational leadership and teacher development allows me to assess the pedagogical relevance and practical implications of the findings, especially regarding learner engagement and classroom implementation. However, my assessment does not extend to detailed neuroscientific validation of music–language processing, which I consider only at a conceptual level.

I confirm that I have read this submission and believe that I have an appropriate level of expertise to confirm that it is of an acceptable scientific standard.

# Reviewer report: Reviewer response for version 1

**DOI:** 10.5256/f1000research.187738.r430279

**Published:** 2026-03-04

**Authors:** Jamal Assadi

**Affiliations:** 1 Academic College for Teacher Education, Sakhnin, Israel

This scoping review provides a broad and well-structured overview of studies (2015–2025) on the use of music to enhance L2 listening engagement and oral fluency, with an additional aim of assessing the transferability of these findings to EMI contexts. The authors follow the PRISMA-ScR framework and employ a clear multi-database search strategy. The narrative synthesis is generally strong, and the review situates its findings within relevant theoretical models (Krashen, Patel, Paivio, among others). The conclusions are appropriate and reflect the current state of research, especially the limited direct evidence for EMI settings.

 However, several points require clarification. Minor inconsistencies occur in the reporting of study counts (e.g., 40 vs. 42 studies), and some citations are misaligned—for example, attributing effects of musical training to Krashen, whose work concerns affective filtering rather than music. There is also occasional repetition that could be streamlined for clarity. Addressing these issues will enhance the precision and coherence of the review.

Is the review written in accessible language?

Yes

Are all factual statements correct and adequately supported by citations?

Partly

Are the conclusions drawn appropriate in the context of the current research literature?

Yes

Is the topic of the review discussed comprehensively in the context of the current literature?

Yes

Reviewer Expertise:

English Literature; Teaching/ learning English as a Secod Language. Modern Arab literature, Sufism

I confirm that I have read this submission and believe that I have an appropriate level of expertise to confirm that it is of an acceptable scientific standard.
